# Individual differences in first- and second-order temporal judgment

**DOI:** 10.1371/journal.pone.0191422

**Published:** 2018-02-05

**Authors:** Andrew W. Corcoran, Christopher Groot, Aurelio Bruno, Alan Johnston, Simon J. Cropper

**Affiliations:** 1 Melbourne School of Psychological Sciences, University of Melbourne, Melbourne, Victoria, Australia; 2 School of Philosophical, Historical and International Studies, Monash University, Melbourne, Victoria, Australia; 3 Department of Experimental Psychology, University College London, London, United Kingdom; 4 School of Psychology, University of Nottingham, Nottingham, United Kingdom; Ecole Polytechnique Federale de Lausanne, SWITZERLAND

## Abstract

The ability of subjects to identify and reproduce brief temporal intervals is influenced by many factors whether they be stimulus-based, task-based or subject-based. The current study examines the role individual differences play in subsecond and suprasecond timing judgments, using the schizoptypy personality scale as a test-case approach for quantifying a broad range of individual differences. In two experiments, 129 (Experiment 1) and 141 (Experiment 2) subjects completed the O-LIFE personality questionnaire prior to performing a modified temporal-bisection task. In the bisection task, subjects responded to two identical instantiations of a luminance grating presented in a 4deg window, 4deg above fixation for 1.5 s (Experiment 1) or 3 s (Experiment 2). Subjects initiated presentation with a button-press, and released the button when they considered the stimulus to be half-way through (750/1500 ms). Subjects were then asked to indicate their ‘most accurate estimate’ of the two intervals. In this way we measure both performance on the task (a first-order measure) and the subjects’ knowledge of their performance (a second-order measure). In Experiment 1 the effect of grating-drift and feedback on performance was also examined. Experiment 2 focused on the static/no-feedback condition. For the group data, Experiment 1 showed a significant effect of presentation order in the baseline condition (no feedback), which disappeared when feedback was provided. Moving the stimulus had no effect on perceived duration. Experiment 2 showed no effect of stimulus presentation order. This elimination of the subsecond order-effect was at the expense of accuracy, as the mid-point of the suprasecond interval was generally underestimated. Response precision increased as a proportion of total duration, reducing the variance below that predicted by Weber’s law. This result is consistent with a breakdown of the scalar properties of time perception in the early suprasecond range. All subjects showed good insight into their own performance, though that insight did not necessarily correlate with the veridical bisection point. In terms of personality, we found evidence of significant differences in performance along the Unusual Experiences subscale, of most theoretical interest here, in the subsecond condition only. There was also significant correlation with Impulsive Nonconformity and Cognitive Disorganisation in the sub- and suprasecond conditions, respectively. Overall, these data support a partial dissociation of timing mechanisms at very short and slightly longer intervals. Further, these results suggest that perception is not the only critical mitigator of confidence in temporal experience, since individuals can effectively compensate for differences in perception at the level of metacognition in early suprasecond time. Though there are individual differences in performance, these are perhaps less than expected from previous reports and indicate an effective timing mechanism dealing with brief durations independent of the influence of significant personality trait differences.

## Introduction

The experience of time and its passing is a pervasive, fundamental feature of conscious awareness; one that has occupied experimental psychology as a discipline since its earliest pioneers [1]. However, in spite of the wealth of research attempting to characterise the nature of temporal representation in humans and other animals [2], the underlying neural mechanisms responsible for encoding interval duration remain obscure. The recent proliferation of books, review articles, and special issues dealing with contemporary theoretical accounts of time perception underscores both the substantial achievements that have been made over the past three decades, and the considerable challenges that still remain [3–7].

One of the chief impediments to understanding the perceptual properties of time periods spanning the milliseconds (i.e. ‘subsecond’ intervals) to seconds (i.e. ‘suprasecond’ intervals) range is the extent to which different methodological paradigms give rise to divergent empirical data [8, 9]. Several authors [10, 11] have suggested that such inconsistencies might derive, at least in part, from substantial (but as yet, largely uninvestigated) individual differences in temporal cognition. In the work described here, we use a novel approach towards the measurement of perceived duration that quantifies not only the individual’s perception of short periods of time, but also their insight into the accuracy of such judgements. We then relate these measures to the personality construct of schizotypy as a means of assessing individual differences in performance in the task across a spectrum of personality traits.

### Individual differences in visual duration judgements

Individual differences in perception have recently gained attention as theoretically and experimentally important factors in many aspects of sensory research [12–20], and time perception is no different [10, 11]. Recent research has attempted to identify genetic and neurochemical [21, 22], or structural and morphological [23, 24] factors that predict individual differences in the accuracy and variability of interval timing judgements. Combining behavioural genetics and neuroimaging approaches, Wiener and colleagues [25] found that such differences may be partially attributed to a developmental polymorphism associated with decreased striatal D_2_ receptor density. They suggested that differences in the dopaminergic activation of frontostriatal circuitry, which constitutes a key network in many contemporary models of interval timing [26, 27], might explain between-subject variation in the perception of short durations. First, however, the extent and nature of those individual differences needs to be measured, which we address here.

### Interval timing and personality-based individual differences

A complementary approach to examining various physiological substrates is to measure systematic variability in temporal judgement tasks in relation to self-reported personality traits; a whole-of-system approach. To date, this approach has focused predominantly on personality constructs that are considered to share features with clinical disorders (e.g., schizophrenia and impulse control dysfunctions) thought to involve distorted temporal processing and dopaminergic dysregulation [28, 29]. However, recent efforts to map interval timing variability onto such personality constructs have so far produced mixed results. For instance, Tsai and Yeh [30] found that healthy adults who reported high trait-impulsivity were less sensitive to small differences in durations ranging from 750 to 1350 ms than their low-scoring counterparts, independent of any attentional effects. In contrast, Baumann and Odum [31] and Corvi and colleagues [32] reported no evidence of association between psychometric indices of impulsivity and performance on suprasecond visual duration judgements using temporal-bisection (2s and 4s standards [31]) and temporal-production tasks (60s production and 75s verbal estimation [32]), respectively.

Schizotypy, a multidimensional construct encompassing a variety of experiences and dispositions reminiscent of psychotic-like phenomena [33], is another potential candidate for predicting individual differences in temporal cognition. Originally conceived as a neurobiological susceptibility to schizophrenic disorder [34, 35] schizotypy has been construed more recently as a personality trait that is continuously distributed throughout the general population [36]; one which is not necessarily indicative of latent psychopathology. On the contrary, some trait features might confer certain adaptive advantages and an increasingly common concept in the literature is that of the healthy schizotype and its relationship to creativity [37–40].

This broader, multidimensional conception of schizotypy is particularly appealing in the context of delineating inter-individual differences in temporal cognition. First, its broad scope enables the evaluation of timing variability across diverse populations. Second, mapping systematic timing differences as a function of schizotypal trait expression might reveal useful insights about changes in temporal experience as normal trait variation transitions into psychotic-like disturbance [41]. Finally, the trait’s complex structure (which incorporates a dimension tapping impulsivity) is advantageous for assessing which specific personality components are most relevant for predicting individual differences in duration perception.

Two studies to date have investigated variability of visual duration judgements as a function of schizotypy. Sarkin and collagues [42] reported no difference in the performance of high- vs. low-scoring young adults attempting to reproduce intervals spanning 1 to 25 s. It should be noted, however, that this study involved a coarse-grained classification of participants based on a composite psychometric score. Such aggregate measures are problematic insofar as they may lack sensitivity to between-subject differences that systematically co-vary with a particular trait dimension. More recently, Reed and Randell [43] reported two temporal-bisection experiments in which participants’ ability to correctly categorise probe stimuli as being more similar to either a short or a long reference interval (using two reference pairs of 200/800ms or 300/900ms), was analysed in relation to scores on the Oxford Liverpool Inventory of Feelings and Experiences—Brief Version (O-LIFE(B) [44]). This study revealed significant differences in the apparent duration of these subsecond intervals as judged by high- versus low-scorers on the Unusual Experiences subscale (UnEx) with high-scorers participants tending to categorise probes more accurately than their low-scoring counterparts. No other between-group differences in task performance were detected across the remaining subscales.

Reed and Randell’s [43] results indicate that systematic variation in interval timing might be accounted for by a susceptibility or propensity towards unusual beliefs (e.g., magical ideation) and aberrant perceptual experiences (e.g., hallucinatory phenomena). Alongside evidence that the early visual processing deficits postulated in schizophrenia also extend (albeit in an attenuated fashion) to individuals who score highly on measures of schizotypy [45–47]; that such visual impairments may arise due to temporal processing disturbances [48]; and, finally, that high-UnEx scores predict diminished sensitivity to visual stimuli [15], these data are suggestive of a common linkage between anomalous visual experiences and distorted temporal representations in the contexts of schizotypy and schizophrenia. This motivates us to examine whether healthy adults who are prone to aberrant visual sensations (i.e. score highly on the UnEx dimension of schizotypy) also manifest subtle differences in duration judgements (i.e. the accuracy and variability of interval estimates), as compared to individuals who are less prone to such experiences.

### Measuring confidence in temporal judgment

In addition to investigating whether duration judgements co-vary with profiles of schizotypal trait expression, we also sought to assess the reliability with which individuals were able to evaluate the relative accuracy of their interval estimates. To our knowledge, no previous attempts have been made to quantify variability in individuals’ (second-order) appraisal of their own (first-order) performance on a temporal estimation task. It seems plausible however that differences in timing ability might derive in part from the extent to which individuals have consistent and accurate access to their internal representations of time. The observation that temporal estimation accuracy is improved by the provision of explicit feedback [49] implies that interval timing is modulated to some extent by knowledge of one’s objective timing performance. However, this study showed that participants who performed less accurately on the temporal discrimination task benefited less from feedback than participants who performed more accurately on the task. This finding suggests that differences in the capacity to bring one’s metacognitive or introspective insight to bear on a temporal estimation task might go some way to explaining the variable patterns of interval timing noted across individuals.

### The current study

From a general perspective, we have recently shown how a personality trait-driven approach can be informative in delineating individual differences in the perception of meaning in spatially noisy patterns [15]. Taking a similar approach here, rather than studying personality *per se*, we are primarily interested in examining how individual differences in timing judgments may correlate with an individual’s particular personality traits. We chose schizotypy as a measure of personality for the reasons outlined above, but stress that this study is primarily interested in investigating the extent to which patterns of variance in individual timing performance can be predicted by personality factors, with only a secondary interest in such factors themselves.

In short, this study presents data from two experiments in which a novel variation of the temporal-bisection task was used to investigate whether individual differences in temporal estimation and metacognition can be related to schizotypy. In Experiment 1, we explored the influence of psychophysical factors (stimulus order, stimulus motion, metacognitive feedback) on the estimation of a subsecond bisection-point target, and whether variance in these effects can be explained by self-report measures of schizotypy. We also investigated whether we could enhance the sensitivity of our analysis by increasing the granularity of the response choices availed by the personality measure (i.e. replacing binary response options with a Likert-scale). Experiment 2 builds on these findings by extending the logic of this paradigm to the suprasecond domain. Using schizotypy as a test case, we show how individual differences in first- and second-order timing (i.e. duration estimation and its retrospective appraisal, respectively) can be distinguished and assessed in relation to personality factors.

## General methodology

This section outlines the methodological features that were consistent across both experiments; experiment-specific details are presented in sections 3.1 and 4.1 for Experiments 1 and 2 respectively.

### Participants

Participants were recruited from the Melbourne School of Psychological Sciences’ first-year programme in return for course credit. All participants reported normal (or corrected-to-normal) vision, and no history of neurological or psychiatric disorder. Both studies were approved by the Human Ethics Advisory Group at the University of Melbourne and each participant provided written consent for participation and publication of their (anonymous) data. No individual participated in both experiments.

### Psychophysics

#### Apparatus and stimuli

Visual stimuli were generated using the Psychophysics Toolbox, Version 3 [50] in MATLAB Version 7.10.0 (The MathWorks^®^ Inc., Natick, MA). They were displayed on a calibrated 21-inch Sony Multiscan^®^ G520 CRT monitor (resolution = 1600 x 1200 pixels, frame rate = 100 Hz, mean luminance = 40 cd/m^2^, CIE co-ordinates [x = 0.333, y = 0.377]) within a stationary circular envelope (diameter = 4° of visual angle) located 4° above a central fixation spot. Interval estimates were recorded via a calibrated Cedrus RB-530 response pad (Cedrus Corporation, San Pedro, CA).

#### Psychophysical procedure

**Fig 1:**
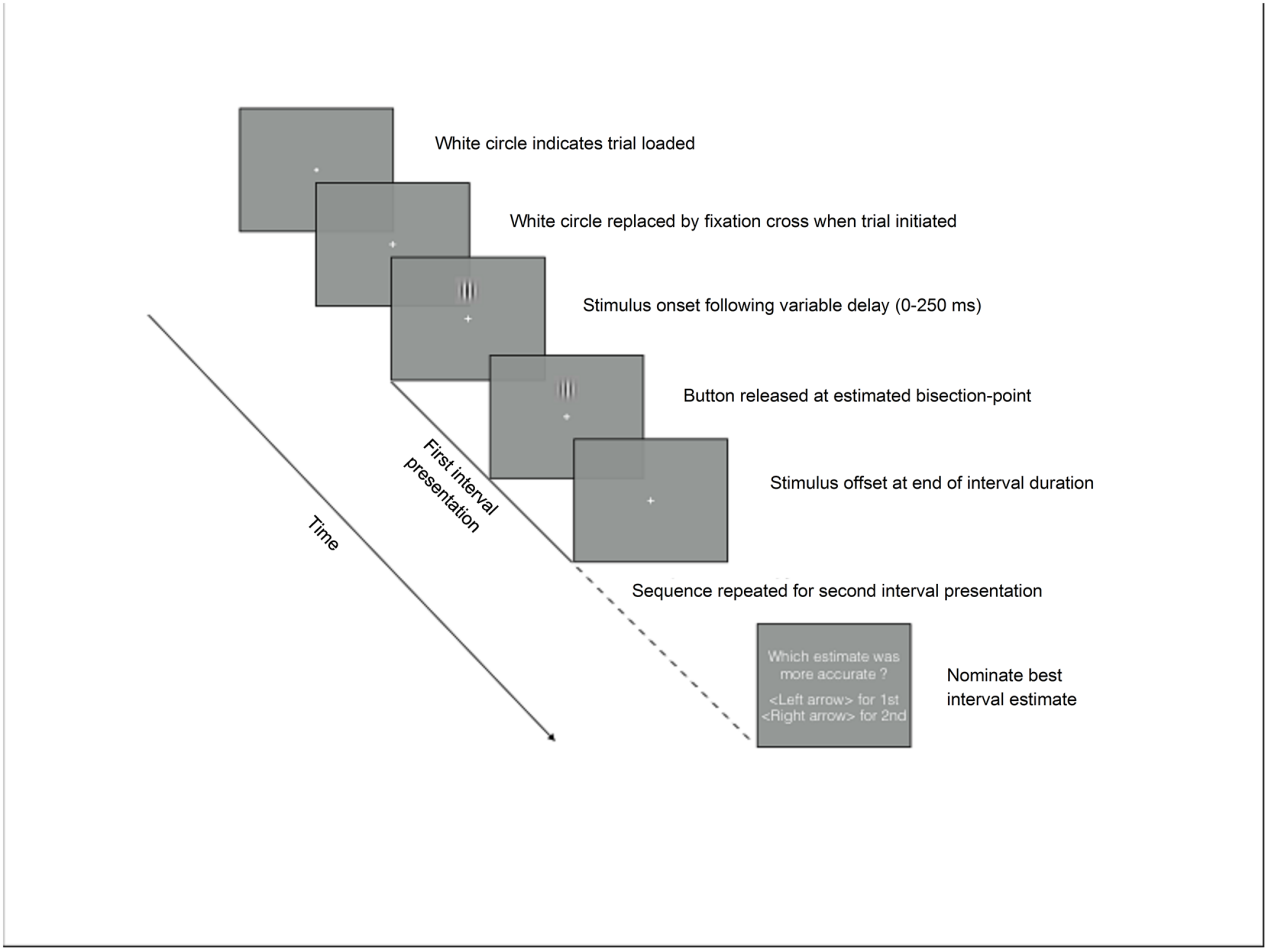
Schematic illustration of the basic sequence of events during a modified temporal-bisection task trial. Duration of interval presentation varied across experiments (1500 vs. 3000 ms). Note, a feedback tone indicating whether the participant had correctly identified which bisection estimate was more accurate was provided at the end of each trial during the Feedback condition of Experiment 1.

We implemented a modified version of a standard temporal-bisection task which was designed to to derive objective measures of first- and second-order temporal judgements in our participants. Each trial involved a (first-order) time estimation component in which participants were required to bisect two intervals of the same duration, and a (second-order) metacognitive appraisal component in which participants were required to identify which of the two preceding bisection estimates was closest to the predefined target duration (i.e. the verdical bisection-point). This hybrid paradigm can, we argue, be thought of as the combination of a temporal-bisection and interval-reproduction task forming the first-order judgement followed by a second-order forced-choice confidence judgement [51]. Since we asked the participants to bisect the interval (through their button-press response) we will describe the data, and the over- or under-estimation of the veridical mid-point of each interval, in terms of a bisection rather than a production. So, for example, if a subject identifies the mid-point of the interval as being prior to the actual mid-point, we consider this to be an underestimation of time in the sense that the whole interval (bisection point x 2) would be experienced as being shorter than it actually is, though this could also be considered to be an overestimation of the *passage* of time in a (re)production scenario. A schematic representation of the generic trial structure is depicted in Fig 1.

All trials consisted of two identical intervals (one of which is represented in Fig 1), each being demarcated through the continuous presentation of the test stimulus; the temporal cue was therefore solely visual. The participant was told the interval duration and that they were required to estimate half of this, bisecting the period that the stimulus was present on the screen. The first interval was initiated when the participant depressed the response pad button. A random delay offset ranging from 0 to 250 ms was inserted after the button press prior to the appearance of the stimulus to avoid the use of rhythm in the task (which we found made it trivial). Upon the appearance of the stimulus, the participant maintained button depression until the interval mid-point, when they released the button. The stimulus remained on-screen until the full duration of the interval had elapsed. This procedure was then repeated for the second interval estimate following a 500 ms inter-stimulus interval.

After making a pair of bisection-point estimates, participants were prompted to make a two-alternative forced choice response via the keyboard arrow keys to indicate which of their estimates they deemed closest to the target duration; their ‘Best’ estimate. This retrospective judgement constituted the temporal metacognition component of the task. This feature of the paradigm allows partitioning of the first-order estimate data according to the participant’s subjective appraisal of their performance on each trial, thus affording an implicit, objective measure of temporal confidence (see section 2.5 for further discussion). It also enabled us to introduce feedback indicating whether the participant had correctly identified the more accurate of their paired estimates. It is only this aspect of the task on which were able, or wanted, to provide feedback on. The feedback does not provide any cue as to the accuracy of the better response *per se* as it can only indicate which of the pair of responses was closest to the veridical mid-point. While this could be used to hone subsequent the estimate after each pair, as we expect and indeed consider an important feature of the paradigm, it does not give a sign or magnitude of ‘error’ cue to the participant.

### Questionnaire materials

Schizotypy was assessed using the Oxford-Liverpool Inventory of Feelings and Experiences (O-LIFE) [52], a 104-item questionnaire consisting of four subscales. The Unusual Experiences (UnEx) scale contains items pertaining to aberrant perceptions, magical ideation, and irrational beliefs (e.g., ‘When in the dark do you often see shapes and forms even though there’s nothing there?’). The Cognitive Disorganisation (CogDis) scale relates to difficulties with attentional focus and decision-making, as well as social anxiety (e.g., ‘Do you often have difficulties in controlling your thoughts?’). The Introvertive Anhedonia (IntAn) scale contains items that reflect preferences for independence and solitude over intimacy and social activity (e.g., ‘Are you much too independent to really get involved with other people?’). The Impulsive Nonconformity (ImpNon) scale taps features of disinhibition, including eccentric and antisocial behaviour (e.g., ‘Do you at times have an urge to do something harmful or shocking?’). We used both the traditional binary yes/no response questionnaire and a modified 1–6 Likert-scale version, splitting the participants into two randomly assigned groups. A secondary aspect of our interest here is to examine the characteristics of using a more graded range of possible responses in the questionnaire (Experiment 1).

### General procedure

Participants attended a single test session at the Melbourne School of Psychological Sciences Vision and Attention Laboratory. Following consent procedures, participants were seated at a computer station and instructed to complete an online version of the O-LIFE at their own pace. This questionnaire required approximately 15–20 minutes to complete. Participants were then taken to a semi-darkened experimental booth (ambient luminance approximately 10 cd/m^2^) to perform the modified temporal-bisection task. This took approximately 45 minutes to complete.

### Data analysis

Statistical analyses were conducted using R version 3.4.0 (R Core Team, 2017). Since standard parametric methods are overly sensitive to outliers and departures from normality [53, 54], robust statistical measures of location and association were favoured. M-estimators based on Huber’s ψ function were selected on account of their higher breakdown point and greater robustness to skewness. These estimators were used in conjunction with percentile bootstrap techniques to calculate 95% confidence intervals. This methodological approach has the added advantages of performing well in the context of heteroscedasticity and small sample size [54].

The general analysis strategy can be decomposed into two stages, each of which examined both the estimation and metacognition components of the task. First, psychophysical performance trends were investigated at the group level. This analysis was designed to explore how various factors (e.g., interval duration, stimulus motion, metacognitive feedback) influenced general patterns of temporal estimation, independent of personality differences. Averages of first and second interval bisection-point estimates, and the variance associated with these estimates, were computed and compared for within-subject order effects using a one-step M-estimator.

To examine group-level trends on the metacognitive component of the task, paired interval estimates were re-sorted into two categories: those estimates the subject classified as being the more accurate of the pair (i.e. closer to the mid-point), and remaining (worse) estimates. These are termed Subjective Best and Subjective Worst hereafter, and each set has a mean and variance associated with it in exactly the same way as the Interval 1 and Interval 2 estimates above do. So, the term ‘Subject Best variance’, for example, refers to the variance of the bisection-point estimates judged to be subjectively better (more accurate) by a given participant for a given condition. Averages of these subjectively sorted bisection-point estimates, and the variance associated with these estimates, were then compared using the M-estimator procedure exactly as above.

Psychophysical data were analysed in relation to individual scores on each of the four O-LIFE subscales in order to examine whether bisection-point estimation and appraisal varied as a function of schizotypal trait expression. Mean and variance estimates were averaged across Intervals 1 and 2 to summarise temporal estimation performance. Mean bisection-point estimates were also transformed into a measure of absolute error (unsigned value of the mean estimated bisection-point subtracted from the actual mid-point). These three summary measures were then assessed for evidence of dependency on each of the four O-LIFE subscales using the percentage bend correlation test, a robust measure of correlation [54]. Both Binary and Likert-scale O-LIFE groups were considered individually for the analysis and indicated as such in the results.

Correlations between O-LIFE scores and the variance of Subjective Best estimates were also calculated to assess whether schizotypal personality dimensions were associated with Best/Worst estimate discrimination precision. Paired interval estimates were then re-sorted according to which estimate was actually closest to the mid-point, and the variance of this ‘Ideal’ best estimate (Ideal Best) calculated. Because this estimate is calculated from the individual estimates of each subject for each condition, this accords well with Ideal Observer Theory and can be accurately expressed as the Ideal Observer estimate [55]. This estimate was then divided by the Subjective Best variance to yield an index of metacognitive acuity (Metacognitive Index), where a value of 1 indicated perfect discrimination performance. This value was also correlated with the O-LIFE subscale scores.

## Experiment 1

Experiment 1 was designed to reveal general performance trends on the temporal-bisection task when the target duration was in the subsecond range (i.e. 750 ms). At the first-order level, stimulus presentation was manipulated in order to assess whether spatially localised stimulus drift systematically affected the magnitude of duration estimates. Dynamic stimuli have been shown to dilate the apparent duration of subsecond intervals as compared to stationary equivalents [56–58]. At the second-order level, external feedback was provided in one condition in order to assess how explicit information pertaining to discrimination accuracy affected performance on the metacognitive component of the task.

As indicated above, we were also interested in assessing whether a Likert-scaled version of the O-LIFE is more sensitive to (and hence, a more robust predictor of) interval timing differences across schizotypal trait profiles, as has been shown in other studies [59, 60]. As in these cited studies, this aspect of our investigation was motivated by both theoretical and empirical concerns. On the one hand, a Likert-scaled response format is more conceptually aligned with the notion of schizotypy as a continuous spectrum than the standard binary format; on the other hand, investigations of alternative psychometric measures of psychosis-proneness have indicated that Likert-scaled response formats enhance sensitivity to individual variation along trait dimensions [61–63]. We present a direct comparison of the two response scales here to justify and facilitate its potential future use. We also analyse critical data in equivalent binary terms in the Supporting Information provided for parity with the literature and to assure the reader that our conclusions based on the Likert- scaled responses are consistent and valid.

### Methodological specifics

129 undergraduate students (90 females) aged 17 to 34 years (*M* = 19.28 years, *SD* = 2.11) were randomly assigned to either a Feedback or a No-Feedback version of the temporal-bisection task. In both conditions, participants were required to bisect 1500 ms intervals presented across 4 blocks of 50 trials. While two blocks featured static stimulus presentations, the remainder consisted of trials in which stimuli appeared to drift horizontally (either left or right, randomised across trials) within the presentation envelope at a temporal frequency of 10 Hz (+/- 0-2Hz). Order of block presentation was counterbalanced. Additionally, participants in the Feedback condition heard a tone at the end of each trial that indicated whether they had correctly nominated the more accurate of their two estimates; no information about the absolute duration of bisection estimates was provided. Participants were supervised while performing 5 practice trials prior to each block to ensure that task instructions were understood.

Participants also completed either a binary (*n* = 66) or Likert-scaled (*n* = 63) version of the O-LIFE. For the (standard) binary format, participants selected either “Yes” (scored as 1) or “No” (scored as 0) in response to inventory items. For the Likert-scaled format, participants rated their agreement with each item on a 6-point scale ranging from 1 (“strongly disagree”) to 6 (“strongly agree”). Scores for each subscale are calculated following the reverse coding of negatively keyed items, with higher subscale scores indicative of higher expression of the schizotypal trait component. The relation between temporal-bisection task performance and personality was investigated separately for each version of the O-LIFE.

Overall, therefore, there were four experimental groups with N≥30 in each group: No-Feedback + Binary O-LIFE, Feedback + Binary O-LIFE, No-Feedback + Likert O-LIFE, and Feedback + Likert O-LIFE. All subjects made temporal judgements on both static and drifting stimuli though the order was randomised between subjects.

This experiment received approval from the University of Melbourne Human Research Ethics Committee (Ethics ID: 1033610.1).

### Results and discussion

Psychophysical data were missing for 3 individuals from the Binary O-LIFE group, hence these participants were excluded from all analyses involving bisection-point estimation. A further 5 participants failed to register a minimum of 50 valid trials (i.e. bisection-point estimates between 0 and 1500 ms), and were therefore also excluded from analysis. Of the remaining 121 participants, 59 belonged to the Binary O-LIFE group (32 Feedback condition); 62 belonged to the Likert O-LIFE group (33 Feedback condition).

#### Psychophysical data

**Fig 2:**
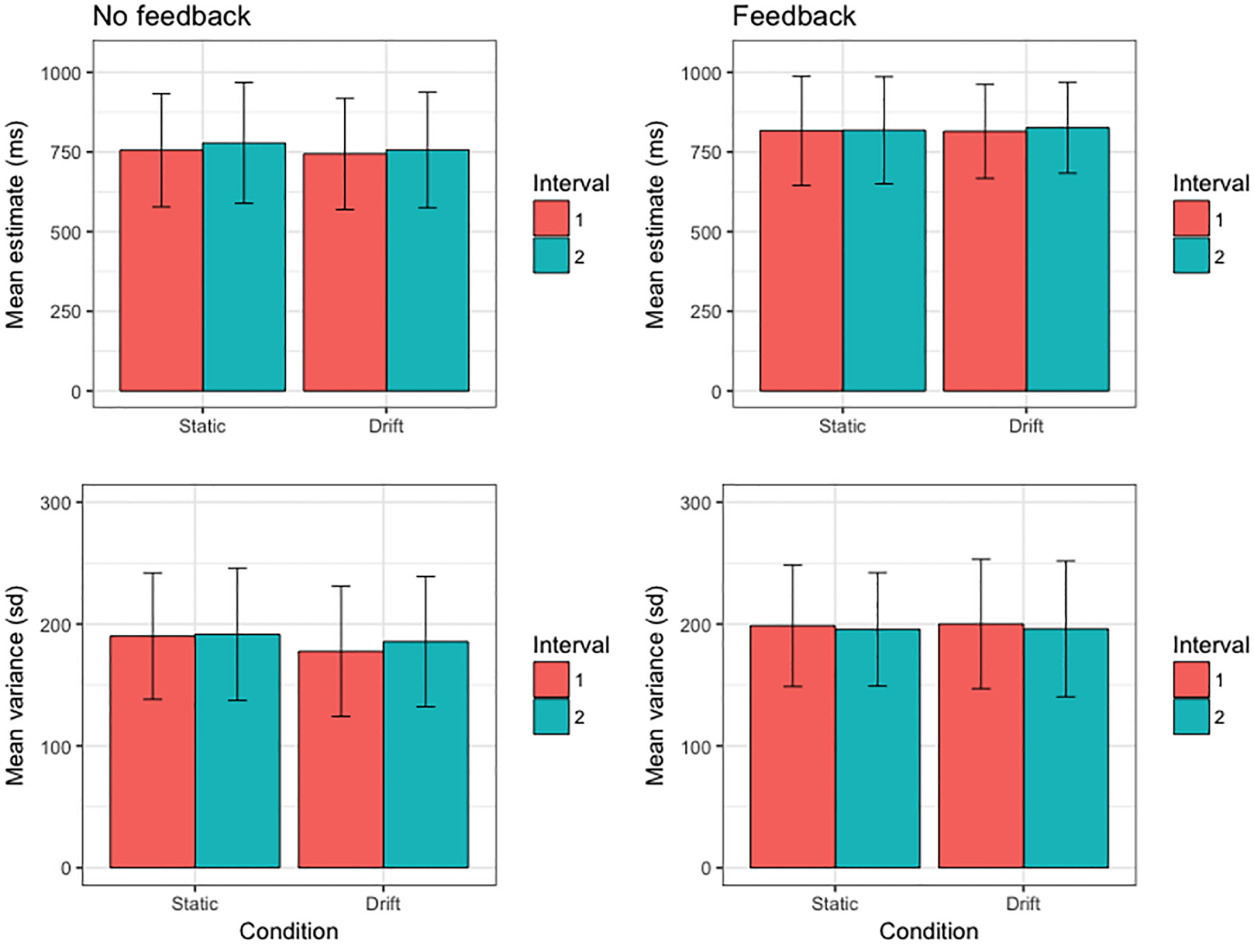
Summary of first-order temporal estimation performance (top row: mean bisection-point duration; bottom row: mean bisection-point variance) across all conditions of the 1500 ms modified temporal-bisection task. Variance data have been transformed into standard deviations for convenience of figure scaling. Error bars represent the standard deviation of the sample mean.

Summary data of group-level task performance are presented in Fig 2. Within-subjects comparisons revealed that Interval 1 bisection-point estimates for static stimuli were on average 26.45 ms shorter than Interval 2 (*p* = .002, 95% CI [10.16, 42.31]). Although contrary to standard accounts of neural coding efficiency, which predict that repeated exposure to identical stimuli elicits diminished neural activity or ‘repetition suppression’ [64], this finding is consistent with previous observations that the first of a pair of duration estimates is experienced as being briefer than the second interval [56, 65]. Interestingly, this effect was eliminated by the provision of feedback (*p* = .722, 95% CI [-10.82, 15.38]), which resulted in elevated (i.e. longer) Interval 1 estimates relative to the No-Feedback condition (*p* = .021, 95% CI [13.31, 130.62]). There were no differences in interval estimate variances within or across feedback conditions (all *p*s ≥ .09).

Bisection-point estimation did not significantly differ as a function of stimulus motion (Static vs. Drift), both in terms of mean duration (all *p*s > .36) and variance (all *p*s ≥ .15). Although this observation seems inconsistent with previous reports of motion (or temporal frequency) induced changes in duration perception, this divergence is most likely attributable to a unique methodological feature of the current paradigm. Unlike previous experiments, which featured either direct (i.e. intra-trial) comparisons [56, 57] or temporal reproductions [57, 58] of static versus drifting stimuli, the bisection task required participants to build up an internal representation of interval duration in relation to stimulus onset and offset. The lack of any discernible within-subject differences in Static vs. Drift condition duration estimates suggests that participants calibrated their estimation of the bisection-point not only in terms of the time elapsed since stimulus onset (as required by the standard method of reproduction), but also in relation to time elapsed between the button release and stimulus offset. As such, any absolute distortion of apparent duration would have been cancelled out by the repeated comparison (and attempted minimisation) of the difference in the duration of the pre- and post-bisection phases of the stimulus interval. This suggestion is consistent with subjective report subsequent to performing the experiment.

**Fig 3:**
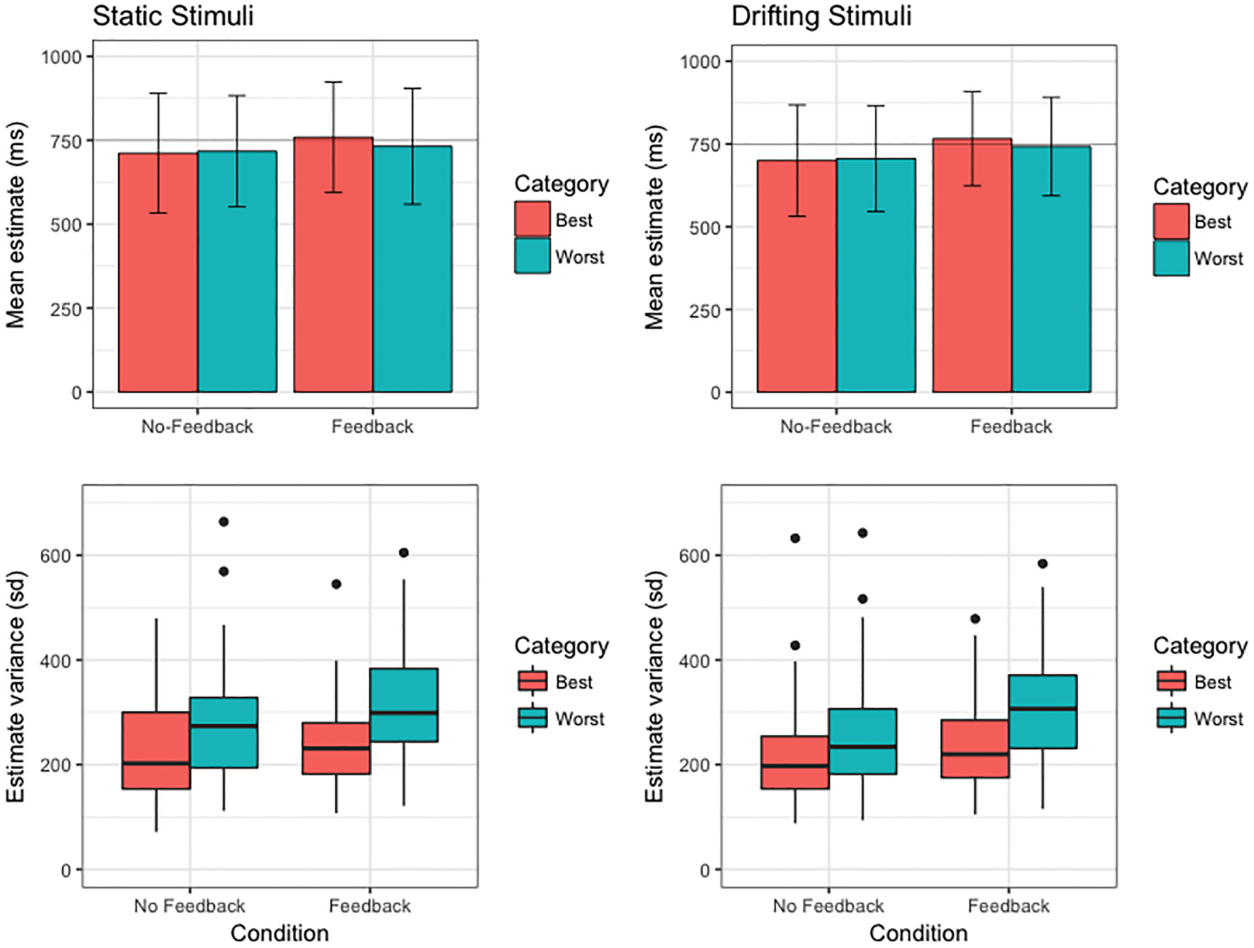
Summary of second-order temporal estimation performance (top row: mean duration of Subjective Best/Worst estimates; bottom row: variance of Subjective Best/Worst estimates across participants) across all conditions of the 1500 ms modified temporal-bisection task. For box plots, thick horizontal line indicates median variance estimate; lower and upper hinges correspond to first and third quartiles, respectively; lower and upper whiskers extend to furthest estimate within 1.5 x interquartile range from the lower and upper hinges, respectively; points indicate outliers. Variance estimates transformed into standard deviations for convenience of figure scaling.

Bisection-point estimate pairs were re-sorted according to the Best/Worst estimate discrimination judgement, and the mean and variance of these estimates re-calculated for each participant. Within-subjects analyses revealed that the mean duration of individuals’ Subjective Best estimates did not significantly differ from that of their Subjective Worst estimates in either No-Feedback condition (*p*s > .07). However, Subjective Best estimates were on average 15.79 ms longer for static stimuli (*p* = .022, 95% CI [1.79, 31.38]), and 18.47 ms longer for drifting stimuli (*p* = .024, 95% CI [2.26, 37.44]), when feedback on discrimination accuracy was provided (Fig 3, top row). This observation is consistent with evidence from the first-order data that feedback on the correctness of their accuracy judgements biased participants towards longer estimate durations.

In line with what we would expect if the participant had insight into their own performance, the variance of Subjective Best estimates was significantly reduced in comparison to Subjective Worst estimates in the No-Feedback conditions (Static: *p* < .001, 95% CI [13311, 24986]; Drift: *p* < .001, 95% CI [10670, 20399]). This finding indicates that participants were generally able to arbitrate the relative accuracy of their paired estimates with some degree of proficiency. The difference between Subjective Best/Worst estimate variances was amplified in the Feedback conditions (Static: *p* < .001, 95% CI [28116, 52687]; Drift: *p* < .001, 95% CI [23288, 41974]), suggesting the precision of temporal metacognitive discrimination judgements is improved when internal representations of duration are supplemented with external information regarding objective timing performance (Fig 3, bottom row).

#### Personality data

**Fig 4:**
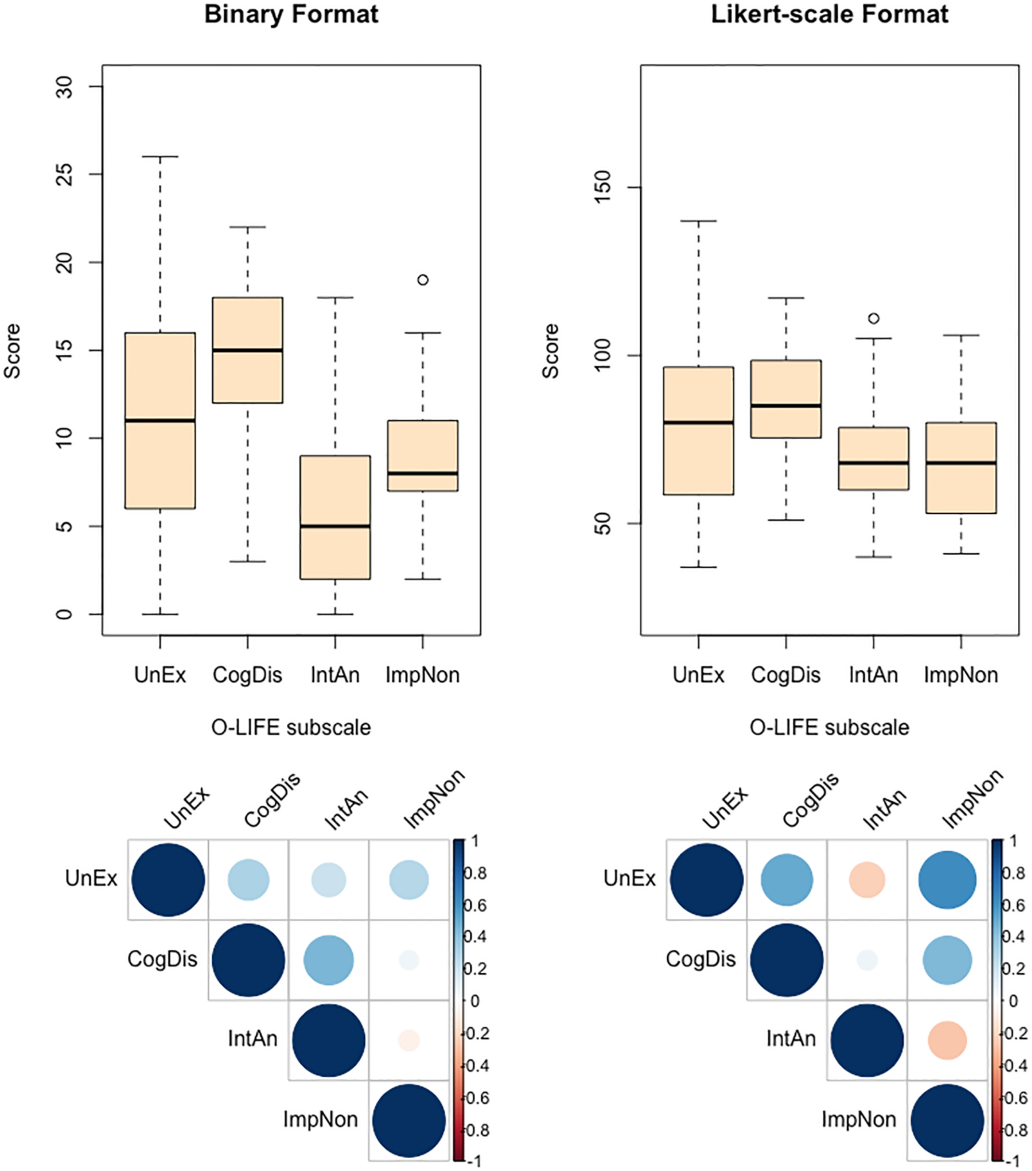
Summary of O-LIFE subscale scores (top row: distribution of subscale scores; bottom row: intercorrelations between subscale scores) as a function of inventory response format. For box plots, thick horizontal line indicates median subscale score; lower and upper hinges correspond to first and third quartiles, respectively; lower and upper whiskers extend to the furthest score within 1.5 x interquartile range from the lower and upper hinges, respectively; points indicate outliers. Y-axes scaled according to range of possible scores afforded by each format. For correlation plots, strength of correlation indicated along Y-axis. UnEx: Unusual Experiences; CogDis: Cognitive Disorganisation; IntAn: Introspective Anhedonia; ImpNon: Impulsive Nonconformity.

Binary O-LIFE scores were broadly comparable to previously reported norms [66]. Likert-scaled responses demonstrated a similar pattern of subscale response distributions and intercorrelations to those reported for the standard format (Fig 4). Interestingly, the Likert-scaled inventory tended to elicit less extreme scores relative to the limits of each subscale. It also tended to amplify the strength of significant intercorrelations between UnEx scores and responses on both the CogDis (*r*_*pb*_ = .50, *p* < .001, 95% CI [.26, .70]) and ImpNon (*r*_*pb*_ = .65, *p* < .001, 95% CI [.47, .78]) subscales. We consider that these are both positive features of the novel modification of the questionnaire. In what follows, analyses of the relation between task performance and O-LIFE subscale scores are reported separately for each O-LIFE response format group.

There was no evidence of association between binary response scores on any of the four O-LIFE subscales and either the mean estimate duration (all *p*s > .35), absolute error (all *p*s > .10), or variance (all *p*s > .06), irrespective of feedback condition. Neither were there any significant correlations with Subjective Best variances in both the No-Feedback and Feedback conditions (all *p*s ≥ .20). The Metacognitive Index was negatively correlated with ImpNon (*r*_*pb*_ = -.41, *p* = .040, 95% CI [-.68, -.02]), suggesting that individuals with higher degrees of impulsivity-related characteristics manifest a reduced capacity to discriminate the more accurate of their paired interval estimates. Metacognition was not significantly associated with any of the remaining three subscales (all *p*s ≥ .13).

The Likert-scaled O-LIFE data likewise provided no evidence of association with either mean estimate duration or estimate variance, irrespective of feedback condition (all *p*s > .19). However, UnEx was positively correlated with the absolute error of the bisection-point estimate in the No-Feedback condition (*r*_*pb*_ = .46, *p* = .010, 95% CI [.12, .76]), suggesting that individuals with higher UnEx scores tended to make less accurate bisection-point estimates when external information concerning the accuracy of their temporal metacognitive judgements was not available. The lack of any clear trend towards either the under- or overestimation of the target duration is consistent with findings in the schizophrenia literature [67]. There was no evidence of any association between UnEx scores and average estimate errors in the Feedback condition (*r*_*pb*_ = .04, *p* = .841, 95% CI [-.32, .39]), which is consistent with the earlier observation that feedback tends to induce additional first-order estimate error as participants attempt to minimise the difference in the duration of estimate pairs. Remaining subscales were uncorrelated with absolute error estimates across both feedback conditions (all *p*s > .17).

**Fig 5:**
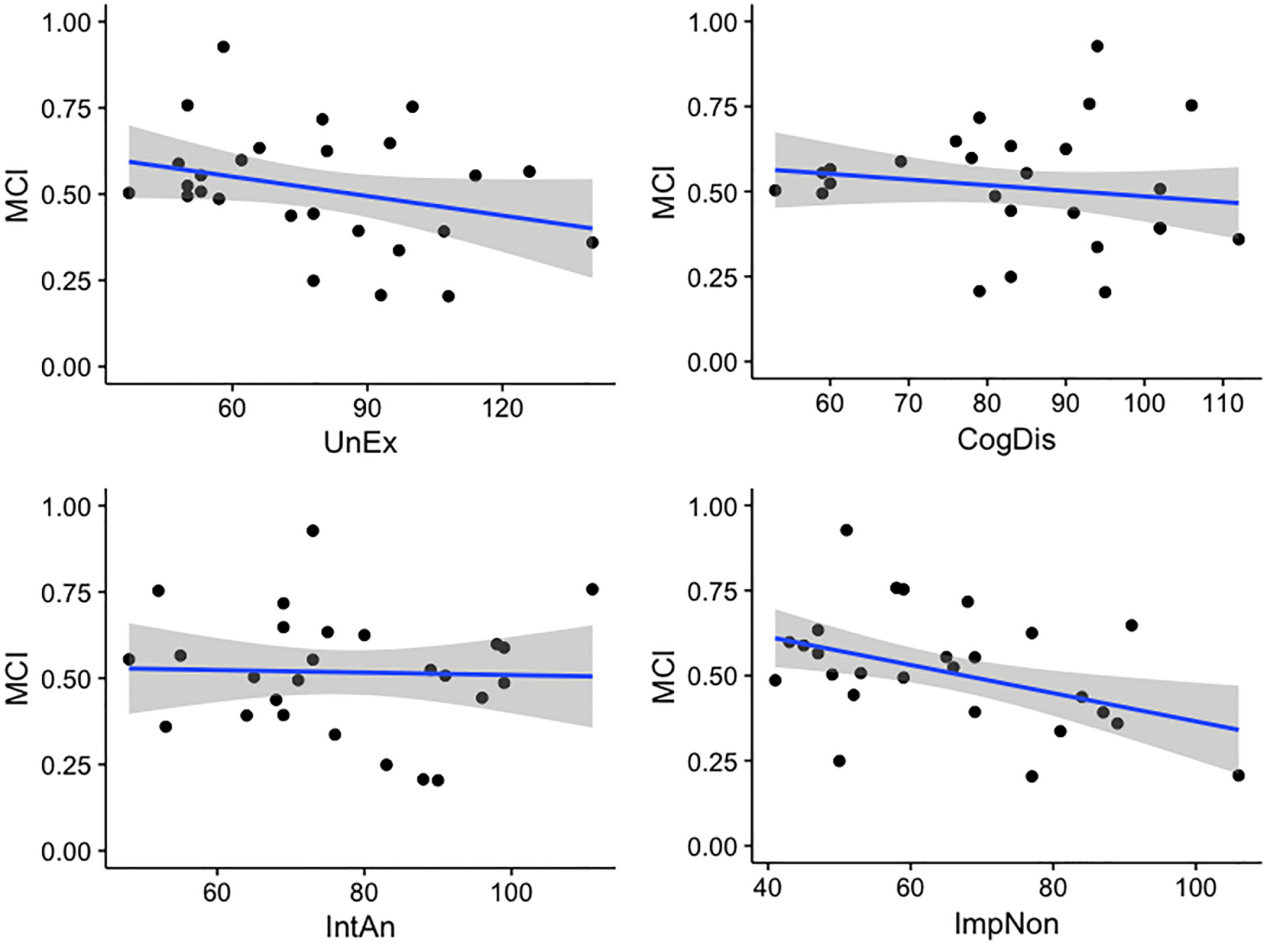
Scatter plots indicating association between O-LIFE subscale scores (Likert-scale response group) and Metacognitive Index score (where 1 corresponds to perfect discrimination of Best/Worst estimates). Blue line indicates robust linear model fit; grey zone indicates standard error of model fit. UnEx: Unusual Experiences; CogDis: Cognitive Disorganisation; IntAn: Introspective Anhedonia; ImpNon: Impulsive Nonconformity; MCI: Metacognitive Index.

Likert-scaled UnEx scores were positively correlated with Subjective Best variances in both the No-Feedback (*r*_*pb*_ = .47, *p* = .027, 95% CI [.09, .74]) and Feedback (*r*_*pb*_ = .35, *p* = .040, 95% CI [.02, .63]) conditions. ImpNon was also positively associated with Subjective Best variance in the No-Feedback condition (*r*_*pb*_ = .43, *p* = .040, 95% CI [.02, .76]) only. Given the absence of any significant correlation between the first-order variance of bisection-point estimates and O-LIFE subscale scores, these findings suggest that greater expression of UnEx and ImpNon characteristics coincide with a reduction in the degree of confidence with which one can introspectively appraise one’s performance on the subsecond bisection task. Evidence of negative correlations between Metacognitive Index and both UnEx (*r*_*pb*_ = -.34, *p* = .077, 95% CI [-.64, .05]) and ImpNon (*r*_*pb*_ = -.37, *p* = .047, 95% CI [-.70, -.01]), albeit at trend level in the former, tentatively corroborate the link between the expression of these trait components and diminished temporal metacognitive sensitivity (Fig 5). By contrast, CogDis and IntAn scores were uncorrelated with both the Subjective Best variance (all *p*s > .12) and Metacognitive Index (all *p*s > .45).

As participant responses on the UnEx and ImpNon scales were highly intercorrelated (*r*_*pb*_ = .58, *p* = .003, 95% CI [.24, .81]), a robust regression analysis was conducted to investigate the extent to which these two scales independently predicted Subjective Best variance. Robust F tests indicated that there was a substantial amount of shared variance between UnEx and ImpNon: while UnEx significantly predicted Subjective Best variance in the No-Feedback condition (*F* = 4.74, *p* = .039), including ImpNon within the model rendered both predictors non-significant (all *p*s > .10). This finding implies that ImpNon subscale scores did not account for a significant proportion of the variance in Subjective Best judgements over and above that accounted for by UnEx scores at this short duration.

#### Summary of Experiment 1

The results reported above reveal that the modified temporal-bisection task is sensitive to group-level order-effects, and that feedback concerning second-order judgement influences first-order estimate behaviour. In contrast, the task was insensitive to previously-demonstrated effects of stimulus motion on temporal estimation. We argue that this latter result arises from the structure of task, suggesting that bisection-point estimates are judged relative to both the beginning and end of each interval.

Feedback increased the average duration of Interval 1 estimates, reducing the discrepancy between estimate pairs. Interestingly, indirect information concerning the relative accuracy of a pair of bisection-point estimates (i.e. whether the estimate that was believed to better approximate the target duration was indeed more accurate) was sufficient to influence first-order estimation performance, biasing subjects towards longer bisection-point estimates. Feedback also served to sharpen the distinction between Best and Worst estimate variance, such that Best estimates where distributed about a much tighter temporal range.

While the binary version of the O-LIFE resulted in virtually no evidence of association between bisection task performance and personality, Likert-scaled responses revealed evidence of selective associations involving the UnEx and ImpNon subscales. These findings are broadly accordant with previous literature implicating psychosis-proneness and impulsivity to altered temporal processing. Interestingly, the modified temporal-bisection task revealed evidence of positive associations between these subscales and second-order estimate variance (Best/Worst, MCI), despite the absence of any consistent association at the first-order level with either under- or over-estimation of the bisection-point, and only UnExp being (positively) correlated with the absolute error. This result speaks to the task’s capacity to uncover dissociations between one’s interval timing performance, and the metacognitive appraisal of one’s performance.

## Experiment 2

The goal of Experiment 2 was to investigate whether trends observed in Experiment 1 could be replicated with a suprasecond bisection-point target (i.e. 1500 ms). Previous studies have indicated that the neuroanatomical bases and neurocognitive mechanisms underpinning suprasecond interval timing are (at least partially) dissociated from those mediating subsecond temporal judgements [24, 68, 69], so it makes sense to examine whether the pattern of results observed in Experiment 1 extend into the suprasecond domain.

In this experiment, all participants completed the Likert-scaled O-LIFE and the Static/No-Feedback condition of the modified temporal-bisection task. We focused on this condition exclusively in order to maximise power, since longer interval estimates are associated with greater variability on account of the scalar property of duration perception according to Weber’s law for time, which stipulates a linear increase in estimate variability as a function of temporal magnitude [70]. We are confident the Likert-scaled version of the O-LIFE provides a usefully more nuanced version of the binary-scale while maintaining fundamentally the same characteristics. Furthermore, since Experiment 1 indicated that our modified version of the temporal-bisection task is insensitive to motion-induced changes in duration perception, there was no rationale for repeating this manipulation.

### Methodological specifics

141 undergraduate students (94 females) aged 17 to 41 years (*M* = 19.37 years, *SD* = 3.09) participated in Experiment 2. All participants completed the Likert-scaled version of the O-LIFE prior to the psychophysics. The stimuli and test conditions for the suprasecond task were similar to those described for the Static/No-Feedback condition of Experiment 1, with the exception that the stimulus was presented for a duration of 3000 ms rather than 1500 ms. The task was again divided into four self-paced blocks of 50 trials. Participants performed 5 supervised practice trials prior to commencing the task.

This experiment received approval from the University of Melbourne Human Research Ethics Committee (Ethics ID: 1339962.2).

### Results and discussion

All participants produced a sufficient number of valid temporal estimates for inclusion within the analysis.

#### Psychophysical data

Although within-subjects comparisons of mean bisection-point estimates indicated that Interval 1 durations were on average 6.62 ms shorter than Interval 2, this difference was non-significant (*p* = .206, 95% CI [-16.66, 3.42]). Within-subjects comparisons of estimate variances were also non-significant across Intervals 1 and 2 (*p* = .274, 95% CI [-1106, 4502]), as per Experiment 1.

**Fig 6:**
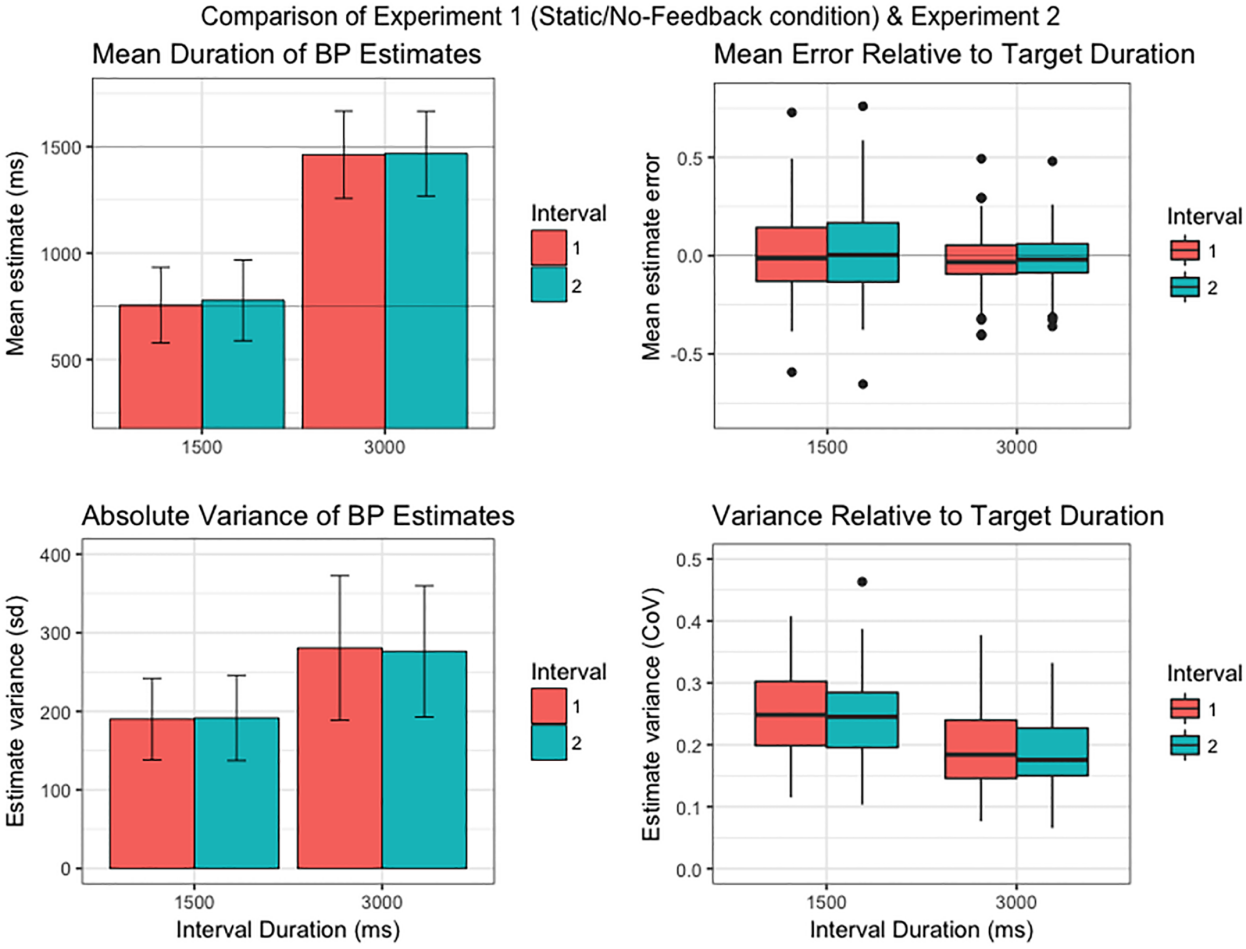
Comparison of first-order timing performance on the suprasecond (3000 ms) modified temporal-bisection task with the homologous condition of the subsecond (1500 ms) task in Experiment 1. Left column: Mean duration (top panel) and variance (bottom panel) of bisection-point estimates across experiments. Bar chart error bars indicate standard deviation of the sample mean. Right column: Mean estimate error (top panel) and variance (bottom panel) relative to target duration. For box plots, thick horizontal line indicates median estimate; lower and upper hinges correspond to first and third quartiles, respectively; lower and upper whiskers extend to furthest estimate within 1.5 x interquartile range from the lower and upper hinges, respectively; points indicate outliers. See main text for details.

Referring to Fig 6, participants undertaking the suprasecond task tended to underestimate the target duration (top left panel), and demonstrated relatively less variability (as a proportion of target duration; i.e. (estimate duration / mid-point target) -1) in the spread of their mean interval estimates (top right panel), than participants completing the subsecond task. Additionally, although variance of interval estimation was clearly elevated in the suprasecond task (bottom left panel), the extent of this increase (bottom right panel) was less than would be expected on the basis of Weber’s law (which predicts a constant coefficient of variation (i.e. standard deviation of estimate durations / mean estimate duration) across different target durations). While contrary to the widespread assumption that short duration interval timing conforms to Weber’s law, this observation is consistent with a growing body of literature positing the breakdown of the scalar property in the early suprasecond range [71]. Reanalysis of the above comparisons using only the first 2 blocks of data collected during Experiment 2 (i.e. the same number of trials completed in the corresponding condition of Experiment 1) indicated that these effects were not simply the consequence of additional task practice (see Supporting Information).

**Fig 7:**
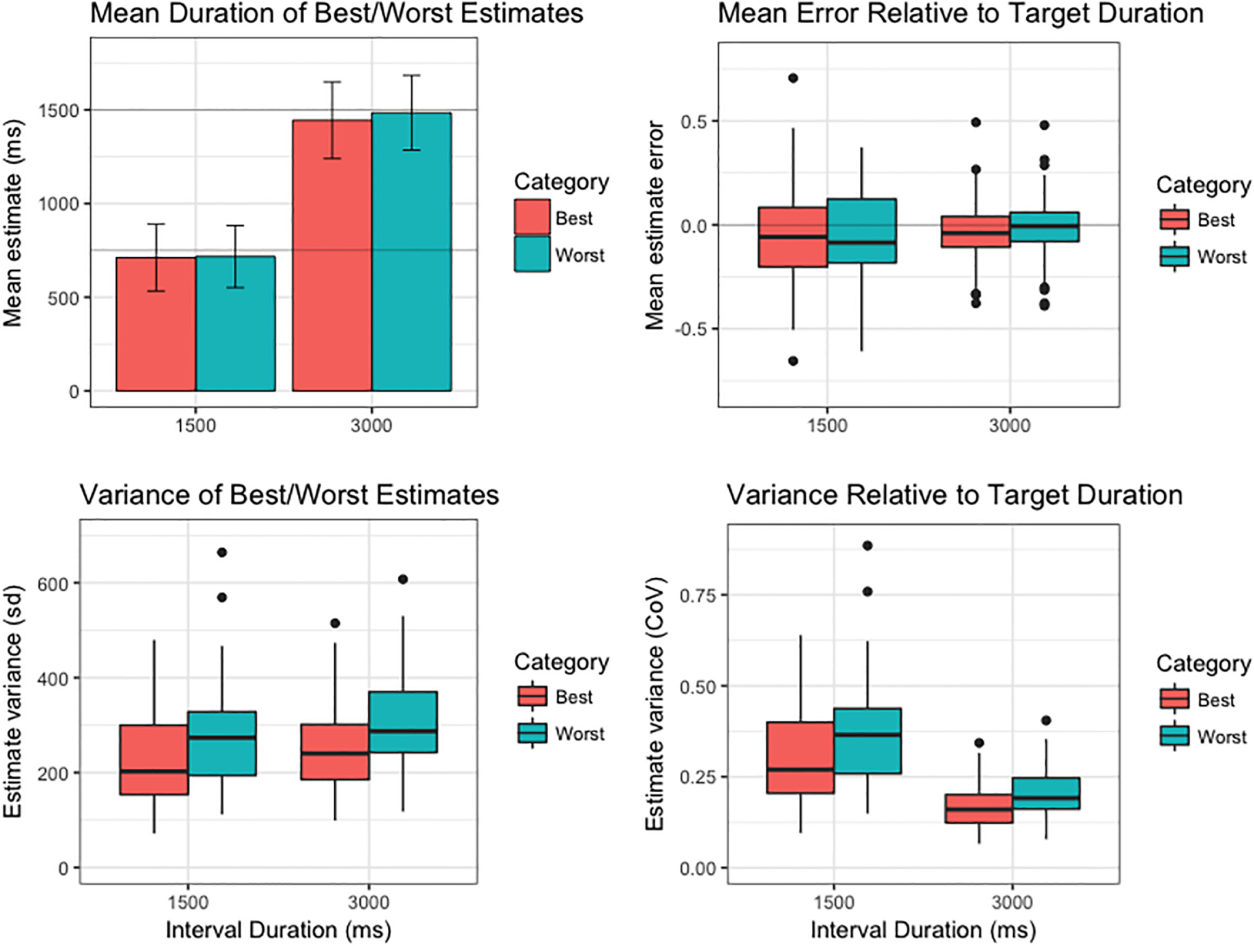
Comparison of second-order timing performance on the suprasecond (3000 ms) modified temporal-bisection task with the homologous condition of the subsecond (1500 ms) task in Experiment 1. Left column: Mean duration (top panel) and group-level distribution of variances (bottom panel) of Subjective Best/Worst estimates across experiments. Bar chart error bars indicate standard deviation of the sample mean. Right column: Mean error (top panel) and variance (bottom panel) of Subjective Best/Worst estimates relative to target duration. For box plots, thick horizontal line indicates median estimate; lower and upper hinges correspond to first and third quartiles, respectively; lower and upper whiskers extend to furthest estimate within 1.5 x interquartile range from the lower and upper hinges, respectively; points indicate outliers. See main text for details.

In contrast to Experiment 1, Subjective Best estimates were shorter than Subjective Worst estimates by an average of 37.80 ms (*p* < .001, 95% CI [26.42, 50.38]). Since Subjective Worst estimates tended to be closer to the target duration, this finding is indicative of a group-level bias in favour of objectively shorter interval durations (corresponding to a dilation of apparent duration). As in Experiment 1 and in line with expectations, Subjective Best estimates were on average markedly less variable than Subjective Worst estimates (*p* < .001, 95% CI [23017, 29985]; Fig 7, bottom left panel). The relative spread of group variability (as indexed by the coefficient of variation) was reduced for both interval categories in comparison to the subsecond task (Fig 7, bottom right panel), which follows from the reduced degree of first-order estimate variability noted above.

#### Personality data

**Fig 8:**
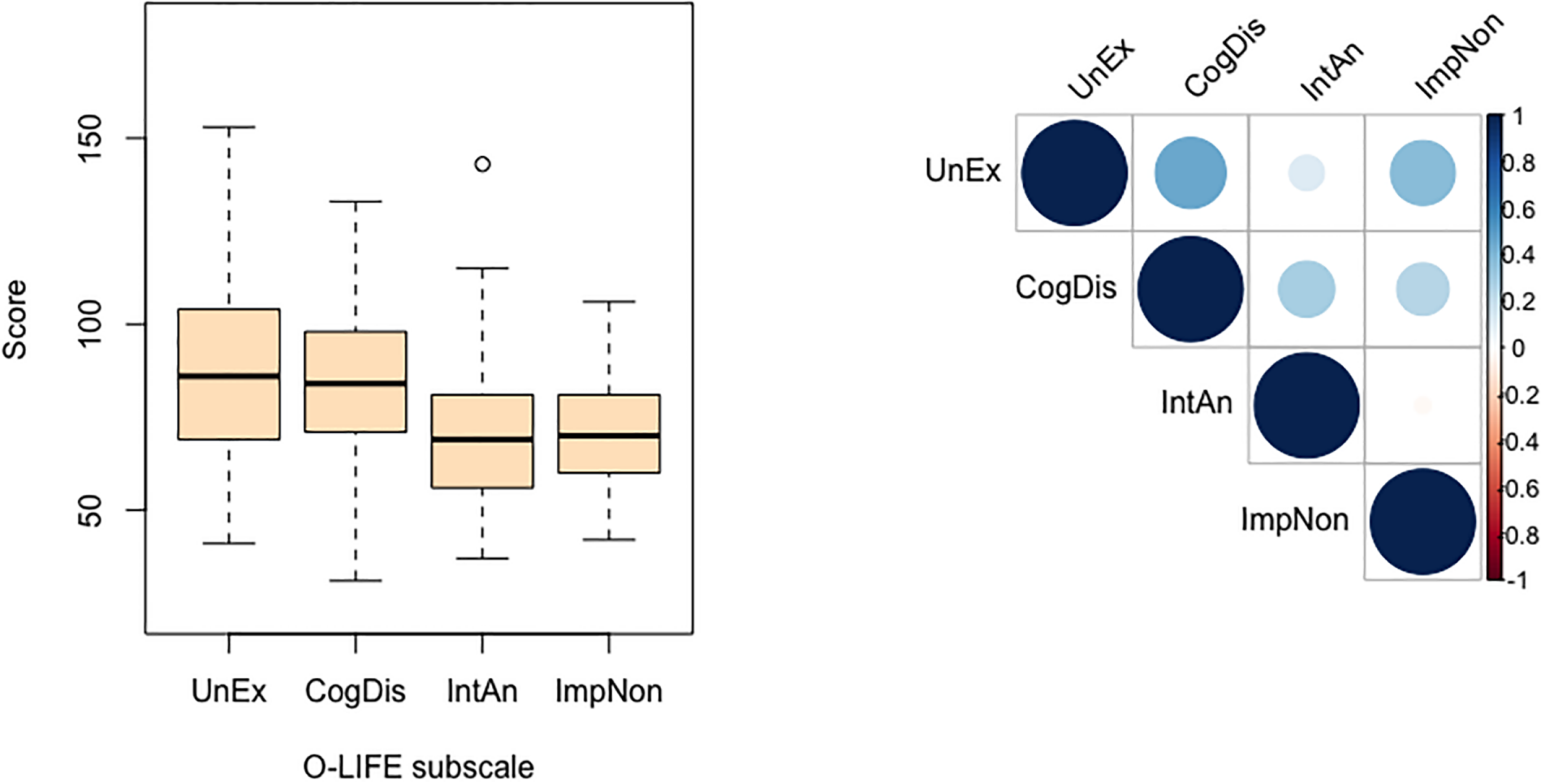
Distribution (left panel) and intercorrelation (right panel) of O-LIFE subscale scores in Experiment 2. For box plots, thick horizontal line indicates median subscale score; lower and upper hinges correspond to first and third quartiles, respectively; lower and upper whiskers extend to the furthest score within 1.5 x interquartile range from the lower and upper hinges, respectively; points indicate outliers. For correlation plots, strength of correlation indicated along Y-axis. UnEx: Unusual Experiences; CogDis: Cognitive Disorganisation; IntAn: Introspective Anhedonia; ImpNon: Impulsive Nonconformity.

Scores on the UnEx scale tended to be higher, and CogDis scores more broadly distributed, than those registered in the Likert-scaled O-LIFE administered in Experiment 1. Response distributions across the IntAn and ImpNon scales were similarly distributed across both groups (Fig 8, left panel). Positive correlations between UnEx and CogDis (*r*_*pb*_ = .41, *p* < .001, 95% CI [.26, .55]), UnEx and ImpNon (*r*_*pb*_ = .34, *p* < .001, 95% CI [.19, .47]), and CogDis and ImpNon (*r*_*pb*_ = .23, *p* = .010, 95% CI [.07, .39]) (Fig 8, right panel) replicated those found in Experiment 1 and mirror reported norms well [66]. A significant correlation between CogDis and IntAn also emerged (*r*_*pb*_ = .26, *p* = .007, 95% CI [.09, .40]). We include a comparison of O-LIFE responses across experiments with all data converted into binary format in the Appendix as a check of this likert-scored version of the questionnaire.

O-LIFE subscale scores were not associated with either mean estimate duration (all *p*s > .18) or absolute error (all *p*s > .61). Only CogDis trended towards positive correlation with estimate variance (*r*_*pb*_ = .15, *p* = .060, 95% CI [0, .31]; all other *p*s > .17). CogDis was also positively associated with Subjective Best variance (*r*_*pb*_ = .18, *p* = .010, 95% CI [.02, .33]); no other scale demonstrated evidence of a substantive relationship (all *p*s > .17). Proportional variance associated with Best/Worst estimate discriminations (Metacognitive Index value) was uncorrelated with any of the four subscales (all *p*s > .43). This latter result suggests that the positive association noted between CogDis and Subjective Best variance most likely issues from a general tendency towards increased estimate variability (note the similarity of the estimated coefficients and confidence intervals), rather than any substantive difference in the nature of temporal metacognitive judgement.

Given the interesting possibility, raised by one of our referees, that the effects observed in the Likert-scale group from Experiment 1 were driven by the conjunction of UnEx and ImpNon trait characteristics, and that analogous effects may have been masked in Experiment 2 by the inclusion of individuals who did not show similar degrees of UnEx and ImpNon covariance, we conducted a subgroup analysis focusing on these two scales. We selected participants by calculating median splits of UnEx and ImpNon scores, and retaining only those individuals who fell the same side of the median on both subscales. This procedure resulted in a subgroup of 87 participants whose UnEx and ImpNon scores were strongly positively correlated (*r*_*pb*_ = .83, *p* < .001, 95% CI [.78, .88]). Repeated analyses revealed no evidence that the effects obtained in Experiment 1 were replicated in this subgroup (all *p*s > .42), indicating that the confluence of UnEx and ImpNon trait expression did not significantly modulate suprasecond temporal judgement.

## General discussion

The two experiments reported here present an analysis of group-level performance and personality-based differences in the ability to bisect brief temporal intervals. In general, participants learned to perform the task relatively well; very few individuals were excluded from analysis due to an insufficient quantity of valid estimates, and most individuals showed some degree of proficiency in appraising the accuracy of their estimates. Although the subsecond bisection task revealed evidence of systematic differences in both the first- and second-order temporal judgments of individuals who reported higher degrees of Unusual Experiences and Impulsive Nonconformity, these effects were not evident in the suprasecond task. Furthermore, Experiment 1 also provided evidence that the Likert-scaled version of the O-LIFE may provide a more sensitive measure of schizotypal characteristics (or at least, of the trait features that predict timing differences) in healthy young adults compared to the standard (binary) response format. This is consistent with the conclusions arising from a series of studies in insight and problem solving [59, 60] and other work on hallucination-proneness ongoing in the lab.

In the discussion that follows, implications of the group-level psychophysical data are dealt with first, followed by our interpretation of the results concerning schizotypy and temporal judgement.

### Psychophysical data

The time-order effect observed during the No-Feedback condition of Experiment 1 replicated earlier reports of what Fechner called ‘negative error’ [65]; i.e. the tendency to underestimate the first of a pair of magnitude estimates. The absence of this effect in the Feedback condition also lends credence to Jamieson and Petrusic’s [65] assertion that prior studies had failed to elicit this effect on account of the feedback information participants were provided while performing the task. Their suggestion that feedback encourages the adoption of biased decision criteria seems consistent with the present findings, insofar as the introduction of feedback in Experiment 1 abolished the order effect at the expense of first-order estimate accuracy (which on average moved further away from the actual target bisection-point). The modulation of first-order interval timing behaviour by external information pertaining to the accuracy of second-order temporal judgements (rather than direct feedback concerning the accuracy of first-order estimates which we did not provide) supports our hypothesis that beliefs about one’s temporal experience (i.e. metarepresentations of time) are capable of influencing the underlying mechanisms involved in the processing of duration perception.

The lack of any systematic time-order effect in the suprasecond bisection task is, however, inconsistent with Jamieson and Petrusic’s [65] study, which obtained analogous effects in the suprasecond domain. Since the 3000 ms interval bisections reported in Experiment 2 were relatively more consistent than those of the 1500 ms duration when absolute magnitude is taken into account (see Fig 6), this result does not appear to be an artefact of greater estimation variability deriving from the scalar property. One possibility is that participants performing the suprasecond task found it easier to compare the time that elapsed either side of the button release, on account of the longer durations of these interval segments. That is, participants may have benefitted from being able to integrate a relatively higher proportion of the temporal information encoded in the suprasecond stimulus presentation as compared to the subsecond stimulus presentation, thus enabling them to regulate their subsequent estimates more effectively. In any case, we note that repetition effects are complex and heterogeneous phenomena, insofar as various interacting (and sometimes opposing) factors can influence the extent to which stimulus repetition suppresses (or enhances) neural responses[3, 72]. We argue that the modified temporal-bisection task presented here offers an ideal paradigm for exploring the subtle nuances of visual stimulus repetition effects on the experience of duration.

### Personality data

Timing performance was correlated with O-LIFE subscale scores to investigate whether distinct profiles of schizotypy are associated with different patterns of duration estimation. On the basis of previous research [15, 43], we were particularly interested in whether the Unusual Experiences (UnEx) subscale could be used to identify distinct patterns of temporal judgement.

The Likert-scaled O-LIFE data from Experiment 1 were in support of the hypothesised relation between UnEx and temporal judgements. Increased UnEx scores were associated with less accurate estimates of the subsecond bisection-point, suggesting this feature of schizotypy shares some degree of continuity with the interval timing disturbances reported in schizophrenia [73, 74]. However, this finding cannot be straightforwardly attributed to a noisier visual processing stream per se, since these estimates were not systematically associated with greater variability (as compared to individuals with lower UnEx scores). While high-UnEx individuals might encode subsecond durations differently compared to others, this difference appears to be relatively stable or consistent (at least within a single test session).

The additional observation that high-UnEx was associated with the diminished precision of temporal metacognitive judgements, which persists even with the provision of performance feedback, supported the hypothesis that patterns of first- and second-order temporal judgements differ along the UnEx dimension. This finding suggests that high-UnEx individuals may be less confident about the veridicality of their subjective experience of time, despite evidence that their estimates of elapsed subsecond durations are on average no less precise than those of others. This observation is consistent with evidence that college students who score highly on measures of schizotypy complain of significant cognitive deficits, despite demonstrating broadly normal levels of performance across a variety of cognitive domains [75].

The lack of any analogous relationship between UnEx and suprasecond timing performance might be accounted for by several explanations. First, it is of course plausible that no relationship exists between these variables in the suprasecond domain, and that the results of Experiment 1 are a product of largely independent subsecond timing mechanisms (this would be consistent with the combined findings of Reed & Randell, [43] and Sarkin et al., [42]). This explanation would suggest that individuals who show high degrees of UnEx might also be expected to manifest subtle perturbations in other domains associated with the same underlying (most notably, cerebellar [69]) networks, such as perceptual decision-making and motor co-ordination [76, 77]

An alternative explanation is that subtle differences in temporal estimation and metacognition were not detected in Experiment 2 due to compensatory strategies enlisted by high-UnEx participants. Given that suprasecond duration estimation is considered to be much more dependent upon mechanisms such as attention and working memory than subsecond interval timing [24, 69, 78], and that these neurocognitive functions are generally intact in university students with high positive schizotypy scores [75, 79], it is possible that temporal processing differences were obscured by use of cognitive strategies that augmented timing performance (e.g., counting, increased attention to temporal information encoded within task stimuli, etc). Indeed, this could explain why the only association between personality and task performance was limited to the Cognitive Disorganisation (CogDis) dimension, since higher degrees of CogDis might translate to the less effective deployment of such cognitive resources. One obvious way to test this hypothesis in future studies would be to introduce an additional task component that increases cognitive load (e.g., a concurrent ‘distracter’ task), thus mitigating against the recruitment of such performance-optimising strategies.

Another potential explanation for the differences observed in the two experiments is that timing differences in nonclinical adults may be more reliably indicated by the conjunction of certain personality features indexed by the UnEx and Impulsive Nonconformity (ImpNon) subscales. It could be the case that the pattern of results found in Experiment 1 was the consequence of sampling a relatively high proportion of individuals who covaried along both of these dimensions. This explanation would go some way to explaining why previous attempts to map interval timing distortions onto psychometrically-defined impulsivity [30–32] have so far produced mixed results. It may be the case that timing differences amongst nonclinical adults are at their most apparent when trait impulsivity co-occurs with the anomalous perceptual experiences and/or patterns of thought tapped by the UnEx scale (e.g., if certain patterns of midbrain/frontal dopaminergic tone underlie the emergence of these personality features as well as temporal processing disturbances). Recent research involving latent profile analysis has indicated that neurocognitive functioning [80] and subjective wellbeing [81] are predicted by specific combinatorial patterns of schizotypal trait expression, suggesting that the link between personality traits and cognitive performance are very likely to be complex and nonlinear.

This being said, the findings of Experiment 2 suggest that the confluence of UnEx and ImpNon trait expression was not associated with analogous differences in temporal judgements in the suprasecond domain (at least for the 1500 ms target duration investigated in this study). If the co-occurrence of UnEx and ImpNon trait characteristics do indeed index a common underlying mechanism that explains individual variance in temporal judgement, this subtle effect would appear to be limited to the subsecond domain. This observation suggests to us that the duration of the temporal interval (i.e. sub- vs. suprasecond), rather than personality dimension combinatorics, is a more substantive source of interindividual differences in timing performance and metacognitive accuracy in the context of trait schizotypy.

### Metacognitive appraisal of estimate accuracy

Both experiments provided strong evidence that participants were able to discriminate what they took to be their best estimate of the target bisection-point with some degree of consistency across trials. The ability to sort paired interval estimates according to the participant’s perception of time and to genuinely apply Ideal Observer analysis is one of the most valuable aspects of the modified task. This feature of the paradigm enables analysis of the subset of temporal estimates which participants consider to be the closest correlates of their own internal representation of the target duration, thus filtering out extraneous variance introduced by motor response errors and other sources of noise. Furthermore, we argue that partitioning estimate variance in accordance with the participant’s metacognitive classification of their Best/Worst estimates gives an implicit, objective index of the confidence with which they are able to introspectively evaluate their own interval timing performance. This is to say that those participants whose Subjective Best estimates fall within a narrower distribution demonstrate greater consistency in their appraisal of their temporal estimates, while those who are less adept at discriminating which estimates fall closer to their subjective representation of the target duration (or indeed, whose representation of the target duration itself varies more greatly from trial to trial) will manifest greater variability on this index. In line with recent research on the behavioural properties [51, 82–86] and neural representation [87] of confidence and uncertainty we maintain that the development of such objective, behaviour-based measures of performance appraisal are essential for assessing the impact of metacognitive processes on duration perception.

The Subjective Best variance does not depend on how closely the individual’s internal representation of the target interval corresponds with the actual mid-point; rather, it indicates how reliably a given subject bisected the interval over the course of the experiment. It should be remarked however that Subjective Best variance is necessarily bounded by first-order estimate variance, meaning that between-subject comparisons could be biased against those individuals whose baseline level of estimate variability is significantly elevated. We therefore calculated the Metacognition Index to provide an indicator of subjective performance that is proportionally scaled in relation to the individual spread of first-order estimates. Taken together, bisection-point estimate variance, Subjective Best variance, and the Metacognitive Index provide sufficient data to make reasoned inferences about the consistency of an individual’s temporal judgements at both the first- and second-order level, and the extent to which performance on these task components can be dissociated.

The capacity to differentiate (dis)continuities between these factors is illustrated by the following two examples: In Experiment 1, UnEx and ImpNon were indicative of diminished temporal metacognitive precision, despite the absence of any clear association between these schizotypy dimensions and first-order estimate variance. In Experiment 2, CogDis was likewise positively correlated with Subjective Best variance, but the absence of any clear association with the Metacognition Index, together with an almost identical relationship with estimate variability, suggested there was little evidence of diminished temporal metacognitive precision over and above the general tendency towards more variable estimation behaviour.

## Conclusion

The modified temporal-bisection task is a novel paradigm for investigating individual differences in interval timing. This paradigm provides statistical information about the accuracy and variability of paired interval estimates as a function of objective performance and the individual’s subjective appraisal of their performance. This latter component of the task provides an objective measure of temporal estimation confidence, which allows for a nuanced analysis of temporal metacognitive processing. We have attempted to show how this paradigm can be applied to differentiate individual differences in temporal judgements in terms of both duration estimation and metacognition. Taking schizotypy as a test case, we found evidence of a negative association between the Unusual Experiences personality dimension and the accuracy of first- and second-order subsecond timing judgements. We did not however find evidence of a similar effect in a suprasecond version of the task. This pattern of results is consistent with previous studies investigating heterogeneity in temporal judgement as a function of psychometrically-defined schizotypy.

## Supporting information

S1 File Additional statistical analyses as cited in text. (DOCX)

S1 Data Raw data files and analysis. (ZIP)
