# Supplementary material for: Individual differences in first- and second-order temporal judgment
Source: PLoS One. 2018 Feb 5;13(2):e0191422. doi: 10.1371/journal.pone.0191422 (PMC5798768; doi:10.1371/journal.pone.0191422)
Supplement: S1 File — (DOCX) [file pone.0191422.s001.docx]

**Supplementary Information**

This file contains additional data analyses and plots designed to facilitate the comparison of group-level results across experiments.

We compared the distributional and correlational properties of all participants’ O-LIFE scores across experiments (Figure A1). This was achieved by converting all Likert-scaled responses into the binary format (Likert scores ≤ 3 were transformed to 0; scores > 3 were transformed to 1). O-LIFE scores from the Likert-scale subgroup in Experiment 1 were pooled with the binary response subgroup (*n* = 129) and compared with binary-transformed scores from Experiment 2 (*n* = 141). Broadly speaking, both samples demonstrated a similar spread of subscale scores, and consistent patterns of intercorrelation between subscales.

We replotted Figure 6 excluding the final 2 blocks of data collected in Experiment 2 (Figure A2). This enabled us to compare performance across the subsecond and suprasecond versions of the modified temporal-bisection task (Static/No-Feedback condition), while holding the number of trials performed in each task constant. This figure shows that that the general pattern of mean and variance estimates observed in Experiment 2 (notably, the decreased variance of estimate error and variation relative to target duration) did not substantially alter as a result of the additional trials performed in Blocks 3 and 4 (included in the main analysis).


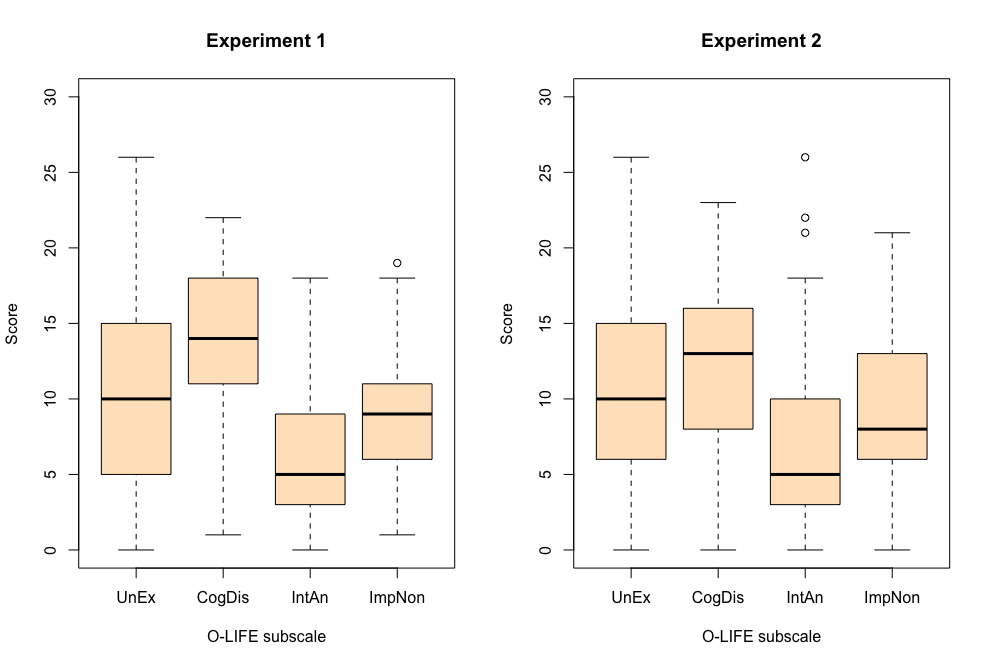


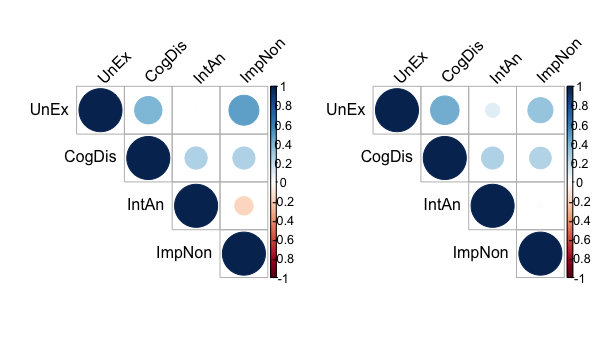


*Fig s1.* Comparison of group-level O-LIFE response profiles (top row: subscale score distributions; bottom row: subscale intercorrelations) across experiments. All responses converted to binary format. For box plots, thick horizontal line indicates median subscale score; lower and upper hinges correspond to first and third quartiles, respectively; lower and upper whiskers extend to the furthest score within 1.5 x interquartile range from the lower and upper hinges, respectively; points indicate outliers. For correlation plots, strength of correlation indicated along Y-axis. UnEx: Unusual Experiences; CogDis: Cognitive Disorganisation; IntAn: Introspective Anhedonia; ImpNon: Impulsive Nonconformity.


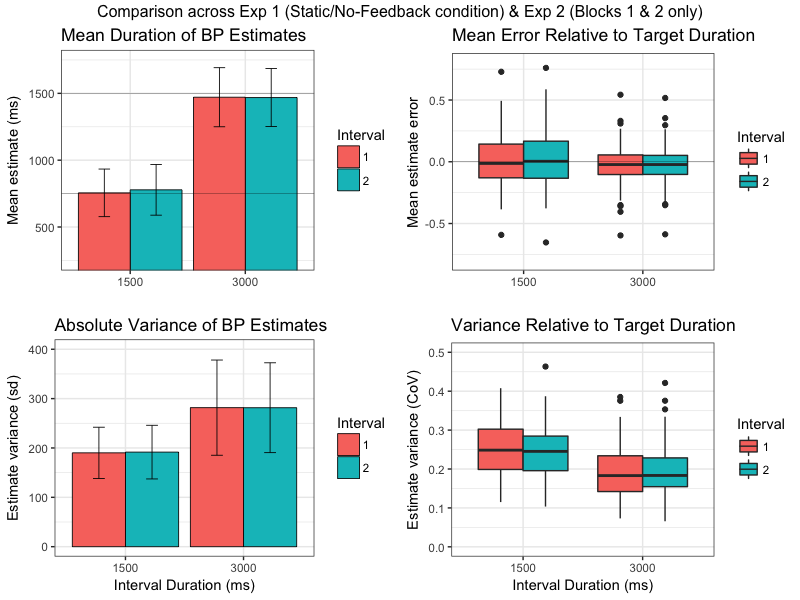


*Fig S2.* Comparison of group-level interval timing performance across experiments. Experiment 1 data (1500 ms interval duration) identical to estimates displayed in Figure 6 (i.e. 100 trials, Static/No-Feedback condition). Experiment 2 data (3000 ms interval duration) are derived from the first 100 trials performed during the task (i.e. Blocks 1 and 2). Left column: Mean duration (top panel) and variance (bottom panel) of bisection-point estimates across experiments. Bar chart error bars indicate standard deviation of the sample mean. Right column: Mean estimate error (top panel) and variance (bottom panel) relative to target duration. Bar chart error bars indicate standard deviation of the sample mean. For box plots, thick horizontal line indicates median estimate; lower and upper hinges correspond to first and third quartiles, respectively; lower and upper whiskers extend to furthest estimate within 1.5 x interquartile range from the lower and upper hinges, respectively; points indicate outliers.
